# Autologous pericardial soft-ring annuloplasty may offer improved long-term control of rheumatic functional tricuspid regurgitation: a propensity-matched cohort study

**DOI:** 10.3389/fcvm.2026.1743805

**Published:** 2026-03-27

**Authors:** Sicong Li, Wei Jiang, Yuyao Qin, Kequan Wei, Hui Chen, Songtao Liu, Xiaomao Long

**Affiliations:** Department of Cardiovascular Surgery, People's Hospital of Guangxi Zhuang Autonomous Region, Nanning, Guangxi Zhuang Autonomous Region, China

**Keywords:** functional tricuspid regurgitation, pericardial annuloplasty, propensity score matching, prosthetic ring, rheumatic heart disease

## Abstract

**Background:**

To compare the long-term efficacy of autologous pericardial soft-ring annuloplasty (P-TVP) versus prosthetic ring annuloplasty (A-TVP) for functional tricuspid regurgitation (FTR) secondary to rheumatic heart disease (RHD).

**Methods:**

A total of 251 consecutive RHD patients undergoing tricuspid annuloplasty from December 2013 to December 2023 were retrospectively analyzed. Among them, 139 underwent P-TVP and 112 received A-TVP. Propensity score matching (1:1) yielded 64 comparable pairs. Follow-up at 6,24,48 and 96 months assessed TR recurrence and right-heart remodeling. Multiple imputation was applied for partially missing follow-up data.

**Results:**

Early outcomes at 6 and 24 months were similar between groups. However, recurrence of moderate TR was significantly lower in the P-TVP group at 48 and 96 months (both *P* < 0.05). Greater right-atrial reverse remodeling was observed in the P-TVP group at 48 months (*P* < 0.05), while right-ventricular diameter remained comparable at 96 months (*P* > 0.05). No increase in procedure-related complications was observed in the P-TVP group.

**Conclusions:**

In this hypothesis-generating study, autologous pericardial soft-ring annuloplasty was associated with more favorable long-term TR control and right atrial reverse remodeling compared with prosthetic ring annuloplasty, despite similar early outcomes. These findings suggest a potential advantage of flexible biological annuloplasty in selected RHD patients, but should be interpreted with caution due to the modest sample size, incomplete follow-up, and retrospective design. Prospective multicenter studies are required to confirm these observations.

## Introduction

Functional tricuspid regurgitation (FTR) is a common valvular disorder, affecting approximately 6.57% of the general population, with most cases being secondary to left-sided valvular heart diseases such as rheumatic mitral or aortic valve lesions (1–3). Progressive pulmonary hypertension and right ventricular (RV) dilation lead to tricuspid annular enlargement and leaflet malcoaptation, resulting in clinically significant regurgitation (4, 5). Without effective intervention, FTR can cause severe right-sided heart failure and is associated with poor long-term prognosis (6).

Surgical repair remains the primary treatment for moderate-to-severe FTR, with annuloplasty being the preferred strategy due to its advantages in preserving the native valvular structure (7). Currently, two main annuloplasty options exist: prosthetic ring annuloplasty and suture-based or autologous pericardial annuloplasty. Although prosthetic rings provide strong annular support and reduce recurrence, they may impair annular dynamics and increase the risk of conduction block or device-related complications (8, 9). In contrast, autologous pericardial annuloplasty avoids foreign-material implantation and may better preserve right-heart biomechanics, but long-term durability remains controversial (10, 11).

Therefore, clarifying the long-term clinical value of autologous pericardial soft ring annuloplasty is of great significance. In this study, we conducted a 10-year retrospective cohort comparison of autologous pericardial soft ring vs prosthetic ring annuloplasty in patients with rheumatic FTR, using propensity score matching (PSM) to reduce baseline bias and multiple imputation to optimize follow-up data completeness. The findings aim to provide high-quality evidence supporting surgical decision-making in FTR.

## Methods and materials

### Ethics statement

This retrospective study was approved by the Ethics Committee of the People's Hospital of Guangxi Zhuang Autonomous Region. The study complied with the Declaration of Helsinki and institutional regulations. Written informed consent was obtained from all participants prior to surgery and data collection.

### Study design and participants

A total of 251 consecutive patients diagnosed with rheumatic heart disease (RHD) and functional tricuspid regurgitation (FTR) who underwent left-sided valve surgery combined with tricuspid valve annuloplasty (TVP) at the People's Hospital of Guangxi Zhuang Autonomous Region between January 1, 2013, and December 31, 2023, were retrospectively reviewed. The inclusion criteria were as follows: (1) Age 18–75 years, either sex; (2) Preoperative echocardiography confirming rheumatic mitral and/or aortic valve disease; (3) Moderate-to-severe tricuspid regurgitation confirmed preoperatively; (4) Complete baseline echocardiographic and electrocardiographic data. Exclusion criteria included:(1) Previous cardiac surgery; (2) Left-sided valve surgery performed without median sternotomy; (3) Coexisting cardiac diseases: coronary artery disease, great vessel disease, or congenital heart disease; (4) Organic/primary tricuspid valve disease; (5) Presence of pacemaker or ICD leads traversing the tricuspid valve.

### Surgical techniques and grouping

**Figure 1:**
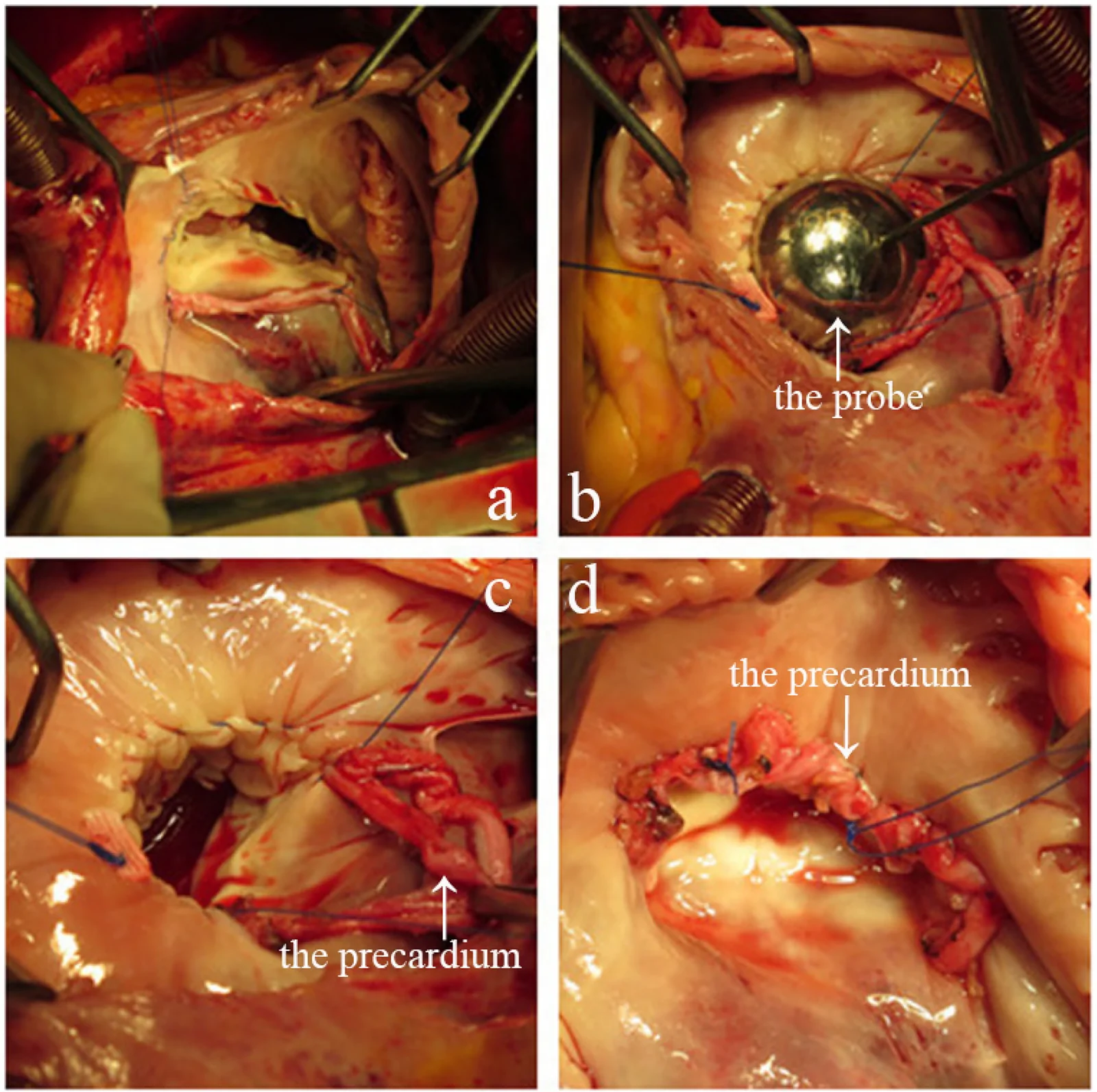
Schematic diagram of P-TVP. **(a)**: Two autologous pericardial strips harvested from the patient; **(b)**: the first suture placement at the upper edge of the coronary sinus; **(c)**: the second suture introduced from the septal annulus at the anterior–septal commissure, followed by clockwise annular suturing perpendicular to the anterior–posterior commissural junction; **(d)**: knotting of the sutures at the anterior–posterior commissure to reduce the annulus to the normal tricuspid size.

Patients were divided into two groups according to the annuloplasty approach: Group A—Autologous pericardial soft ring. Two autologous pericardial strips (0.8 × 10 cm) were harvested, trimmed free of fat, and fashioned into a flexible ring. With 3-0 Prolene mattress sutures, annular plication was initiated at the upper edge of the coronary sinus and progressed clockwise from the anteroseptal commissure. The annulus was reduced according to the appropriate tricuspid valve sizer prior to final knotting. The pericardial strip was then secured continuously along the annulus (Figure 1), with the plication tailored according to the reference sizes provided in Table 1 to achieve a competent and non-stenotic valve. Group B—Prosthetic ring annuloplasty. Patients received a commercially available rigid/semi-rigid prosthetic ring (Bierens model) sized according to standard intraoperative assessment.

**Table 1:** Tricuspid annulus diameter in healthy people (without heart disease).

Number	Body Surface Area (m^2^)	Tricuspid annulus diameter (mm)
1	0.25	13.40
2	0.30	14.90
3	0.35	16.20
4	0.40	17.30
5	0.45	18.20
6	0.50	19.20
7	0.60	20.70
8	0.70	21.90
9	0.80	23.00
10	0.90	24.00
11	1.00	24.90
12	1.20	26.20
13	1.40	27.70
14	1.60	28.90
15	1.80	29.10
16	2.00	30.00

Table 1 shows the results of an epidemiological survey of end-diastolic tricuspid annulus diameter (apical four-chamber view) in 1000 healthy Chinese subjects (inclusion criteria: 18 to 70 years old, both genders, without basic heart disease) in a previous study.

#### Intraoperative sizing for P-TVP

After placing the annular sutures but prior to tying, annular size was assessed using a dedicated sizing probe (Figure 1). The probe, available in diameters of 23, 25, 27, 29, 31, and 33 mm, was selected according to the patient's body surface area as referenced in Table 1. In cases where the dedicated probe was not available, a tricuspid annuloplasty sizer (Bairen, Beijing) was used as an alternative. Following sizing, the sutures were tied. Upon completion of knot tying, initial visual inspection of leaflet coaptation was performed, followed by precise evaluation via transesophageal echocardiography to assess for any residual tricuspid regurgitation or stenosis.

### Echocardiographic assessment

All echocardiographic examinations were performed in the ultrasound department of our hospital. As this study is retrospective, the assessment of TR severity in early patients followed the ASE/EACVI guideline recommendations from the earlier phase of the research, using the maximum regurgitant jet area (cm^2^) measured by color Doppler flow imaging as the primary quantitative index, obtained from the apical four-chamber view. Although subsequent guideline updates (e.g., in 2017) emphasize the comprehensive application of vena contracta width, effective regurgitant orifice area, and regurgitant volume, to maintain data consistency and comparability across all cases in this study, regurgitant jet area was uniformly used for grading.

### Perioperative medical management

#### Preoperative management

All patients received at least five days of diuretic therapy before surgery. The regimen consisted of daily oral administration of furosemide (20 mg), hydrochlorothiazide (25 mg), and spironolactone (20 mg). If the diuretic response was inadequate, intravenous furosemide or torasemide was administered. The treatment goal was to maintain a daily negative fluid balance of 500–1000 mL until systemic congestion signs (e.g., lower limb edema, hepatic congestion) were relieved and the NYHA functional class improved to at least III.

#### Postoperative medical management

All patients received standard anticoagulation therapy with warfarin, targeting an international normalized ratio (INR) of 2.0–3.0 for a minimum of six months. The anticoagulation duration was extended for patients with concomitant atrial fibrillation or mechanical valve implantation. Diuretics were routinely initiated one month after surgery, with subsequent tapering or adjustment based on clinical evidence of volume overload. Management of pulmonary hypertension included oxygen therapy and, when indicated, combination therapy with pulmonary vasodilators (e.g., tadalafil, ambrisentan). Atrial fibrillation was managed according to contemporary guidelines, involving rate control with beta-blockers or digoxin, with consideration for rhythm control using amiodarone or cardioversion.

During follow-up, the same preoperative diuretic regimen was re-instituted for patients who developed severe or greater tricuspid regurgitation.

### Data management and statistics

Baseline demographic and clinical data were collected before surgery. Follow-up evaluations were conducted at 6, 24, 48 and 96 months postoperatively through outpatient visits or telephone interviews, including electrocardiographic rhythm status and echocardiographic parameters (tricuspid regurgitation area, right atrial/ventricular diameters, pulmonary artery systolic pressure, and mitral valve hemodynamics). Missing follow-up data were assumed to be missing completely at random and addressed using five multiple-imputation iterations via predictive mean matching. To minimize baseline differences, propensity score matching [1:1 ratio; caliper 0.05 (The caliper was chosen after testing calipers of 0.02, 0.05, and 0.1, Given the sample size, 0.05 may be optimal)] was performed using age, sex, body surface area, NYHA class, LVEF, pulmonary artery pressure, and atrial fibrillation/flutter as covariates. Statistical analysis was performed with SPSS 29.0 (SPSS Inc, IBM Corp, Chicago, Illinois). Continuous variables were analyzed using independent-sample t-tests and expressed as mean ± standard deviation, while categorical variables were compared using chi-square or Fisher's exact tests and expressed as counts and percentages. A two-tailed *P* < 0.05 was considered statistically significant.

### Handling of missing data

Due to patient relocation, changes in contact information, and loss to follow-up during the study period, some follow-up echocardiographic data were unavailable. In this study, missing data predominantly occurred as the complete absence of follow-up records for individual patients at either the 48-month or 96-month time point. This resulted in situations within successfully matched case pairs where one patient had complete data while the other was missing. Given the value of long-term follow-up data, multiple imputation was employed to handle missing values, aiming to maximize the retention of observational information for each patient.

Imputation was performed using the Multiple Imputation procedure in SPSS Statistics version 29.0 (IBM Corp.). We generated m = 5 imputed datasets to ensure adequate analytical efficiency. The imputation model utilized a Fully Conditional Specification (FCS) approach, with predictive mean matching for continuous variables and logistic regression for binary categorical variables (e.g., atrial fibrillation/flutter, elevated pulmonary artery systolic pressure). The model included the following variables: baseline covariates (age, sex, BMI, treatment group [P-TVP vs. A-TVP]), and all available measurements of key echocardiographic variables from all time points (6, 24, 48, and 96 months) to preserve longitudinal correlations. The process was run for 10 iterations.

Convergence of the multiple imputation algorithm was evaluated by assessing the stability of imputed values across the five independent datasets. For continuous variables, the coefficient of variation (CV) of the mean estimates was used as a reproducibility metric; for categorical variables, variability in positive event rates across datasets was examined.

To examine the potential impact of missing data on study conclusions, we conducted a sensitivity analysis comparing results from multiple imputation with those from a complete-case analysis.

## Results

### Baseline characteristics after propensity score matching

A total of 251 patients were included before matching (139 in the autologous pericardial soft ring group and 112 in the prosthetic ring group). Prior to matching, the prosthetic ring group demonstrated higher proportions of NYHA class IV, severe pulmonary hypertension, and atrial fibrillation (all *P* < 0.05). After PSM, 64 matched pairs (*n* = 128) were generated. No significant differences were observed between the two groups in demographic, clinical, or preoperative echocardiographic variables following matching (all *P* > 0.05) (Table 2).

**Table 2:** Comparison of covariates before and after matching propensity score.

Covariate	Before Matching				After Matching			
	Group A (*n* = 139)	Group B (*n* = 112)	*P*	*SMD*	Group A (*n* = 64)	Group B (*n* = 64)	*P*	*SMD*
Gender (*n*, %)								
Male	43, 30.94%	38, 33.93%	0.640	−0.064	21 32.81%	24 37.50%	0.582	−0.098
Female	96 69.06%	74, 66.07%		0.074	43 67.19%	40 62.50%		0.089
Age, years	51.11 ± 9.50	53.18 ± 9.29	0.082	−0.220	53.39 ± 9.23	51.67 ± 9.36	0.298	0.185
BSA, m^2^	1.53 ± 0.16	1.51 ± 0.17	0.118	0.121	1.54 ± 0.18	1.53 ± 0.17	0.335	0.057
NYHA (*n*, %)								
Ⅱ	9 6.47%	3, 2.68%	<0.05	0.182	2 3.13%	2 3.13%	0.782	0.000
Ⅲ	125 89.93%	78, 69.64%		0.522	58 90.63%	57 89.06%		0052
Ⅳ	5 3.60%	31 27.68%		−0.703	4 6.25%	5 7.81%		−0.061
LVEF, %	57.38 ± 9.40	56.15 ± 7.64	0.150	0.144	56.87 ± 8.10	56.06 ± 7.59	0.559	0.103
PASP (*n*, %)								
Mild	19 13.67%	26 23.21%	<0.05	−0.248	14 21.88%	15 23.44%	0.524	−0.037
Moderate	26 18.71%	25 22.32%		−0.090	24 37.50%	21 32.81%		0.098
Severe	8 5.76%	23 20.54%		−0.449	3 4.69%	8 12.50%		−0.282
Atrial Fibrillation/Flutter (*n*, %)	91 65.47%	86 76.79%	<0.05	-0.252	44 68.75%	51 79.69%	0.157	−0.252

At the 48-month and 96-month time points, the proportion of missing values was 10.46% and 12.50%, respectively. Little's MCAR test indicated that the missingness might not be completely random at both time points (48 months: *χ*^2^ = 16.446, *P* = 0.036; 96 months: *χ*^2^ = 0.00, *P* < 0.001). Therefore, multiple imputation was applied in subsequent analyses to mitigate potential bias arising from the missing data.

Variable selection for propensity score matching was based on clinical importance and the avoidance of multicollinearity. Although preoperative TR severity, RAD and RVD were not directly incorporated into the matching model, these parameters are highly correlated with already matched variables such as NYHA class and pulmonary artery pressure. Furthermore, post-matching comparisons revealed no significant inter-group differences in these parameters (see Table 3), indicating that the matching strategy effectively controlled for potential confounding factors.To comprehensively assess matching quality, we calculated standardized mean differences (SMD) for TR area, RAD, and RVD both before and after matching (Table 3), These results further supports the comparability of the two groups in terms of key baseline characteristics.

**Table 3:** Comparison of major right heart parameters measured by transthoracic echocardiography before and after propensity score matching.

Right Heart Parameters	Before Matching				After Matching			
	Group A (*n* = 139)	Group B (*n* = 112)	*P*	*SMD*	Group A (*n* = 64)	Group B (*n* = 64)	*P*	*SMD*
TR, cm^2^	5.52 ± 5.17	7.67 ± 5.73	<0.001	−0.395	6.17 ± 4.52	5.35 ± 2.29	0.290	0.229
RAD, mm	36.00 ± 8.03	38.96 ± 8.24	0.013	−0.364	37.43 ± 6.94	38.97 ± 7.41	0.226	0.215
RVD, mm	33.27 ± 6.94	34.51 ± 7.15	0.106	−0.176	33.38 ± 6.22	34.95 ± 6.66	0.171	0.244

For the three variables not directly included in the matching process—TR area, RAD, and RVD—the post-matching standardized mean differences were all within an acceptable range (<0.25). This further supports the comparability of the two groups in terms of key baseline characteristics.

After matching, all covariates included in the PSM model demonstrated standardized mean differences (SMD) less than 0.1 between the two groups (see Table 2), indicating satisfactory matching efficacy. For preoperative TR area, RAD, and RVD—variables not directly included in the matching process—the post-matching SMDs were 0.229, −0.XX, and −0.XX, respectively, all within an acceptable range (<0.25), further supporting the comparability of the two groups on key baseline characteristics.

### Convergence and sensitivity analysis of multiple interpolation

Convergence and Model Diagnostics (Table 4): For continuous variables, the coefficient of variation (CV) of the mean estimates was consistently below 2%, indicating high reproducibility. Specifically, at 48 months, tricuspid regurgitation area showed a CV of 1.8%, right atrial diameter 0.3%, and right ventricular diameter 0.4%. At 96 months, all continuous variables remained stable with CVs between 0.5% and 1.5%. For categorical variables, the positive event rates for atrial fibrillation and pulmonary artery systolic pressure grading fluctuated within a narrow range of ±1.1% across imputed datasets. These results demonstrate robust convergence and reliability of the imputation procedure.

**Table 4:** Comprehensive convergence analysis for all variables across multiple imputed datasets.

Time Point	Variable	Metric Analyzed	Range Across Imputed Datasets	Coefficient of Variation (CV*)/Fluctuation Assessment
48 Months	Continuous Variables			
	MVPG (mmHg)	Mean	15.2–15.8	1.2%
	MV(m/s)	Mean	1.96–2.02	1.1%
	PHT (ms)	Mean	101–105	1.3%
	TR area (cm^2^)	Mean	3.47–3.65	1.8%
	RAD(mm)	Mean	34.9–35.2	0.3%
	RVD(mm)	Mean	32.4–32.7	0.4%
	EF(%)	Mean	54.1–54.9	0.5%
	Categorical Variables			
	AF(Yes?)	Positive Rate	48.5%–50.5%	Fluctuation ±1.0%
	PASP (Grade)	Positive Rate	10.2%–11.8%	Fluctuation ±0.8%
96 Months	Continuous Variables			
	MVPG (mmHg)	Mean	16.5–17.1	1.4%
	MV(m/s)	Mean	2.15–2.22	1.2%
	PHT (ms)	Mean	108–113	1.5%
	TR area (cm^2^)	Mean	4.85–4.93	0.7%
	RAD(mm)	Mean	36.5–36.9	0.5%
	RVD(mm)	Mean	32.8–33.3	0.7%
	EF(%)	Mean	53.2–53.9	0.5%
	Categorical Variables			
	AF(Yes?)	Positive Rate	52.0%–54.1%	Fluctuation ±1.1%
	PASP (Grade)	Positive Rate	15.5%–17.0%	Fluctuation ±0.8%

* CV = (Standard Deviation/Mean) × 100%. A CV < 5% indicates excellent stability. For categorical variables, the fluctuation in positive event rates across datasets is shown.*

Sensitivity Analysis (Table 5): Key outcomes—including tricuspid regurgitation area, right atrial diameter, and right ventricular diameter—showed consistent effect directions, comparable magnitude of between-group differences, and concordance in statistical significance between the two analytic approaches. For instance, the treatment effect on tricuspid regurgitation area at 48 months was −1.75 cm^2^ (*P* = 0.002) under multiple imputation and −1.71 cm^2^ (*P* = 0.003) under complete-case analysis. Similar consistency was observed across all continuous and categorical variables at both 48 and 96 months, supporting the robustness of our findings irrespective of the missing data handling method.

**Table 5:** Comprehensive sensitivity analysis: multiple imputation vs. complete-case analysis.

Time Point	Variable	Multiple Imputation (Pooled Results)	Complete-Case Analysis
48 Months	Continuous Variables	Mean ± SD or Between-Group Difference (*P*-value)	Mean ± SD or Between-Group Difference (*P*-value)
	MVPG (mmHg)	15.5 ± 5.1 Diff: −0.8 (*P* = 0.25)	15.4 ± 5.2 Diff: −0.7 (*P* = 0.28)
	MV(m/s)	1.99 ± 0.42 Diff: −0.05 (*P* = 0.40)	1.98 ± 0.41 Diff: −0.04 (*P* = 0.45)
	PHT (ms)	103 ± 28 Diff: −5.2 (*P* = 0.18)	102 ± 27; Diff: −5.0 (*P* = 0.20)
	TR area (cm^2^)	3.56 ± 1.82 Diff: −1.75 (*P* = 0.002)	3.52 ± 1.79 Diff: −1.71 (*P* = 0.003)
	RAD(mm)	35.1 ± 3.2 Diff: −2.1 (*P* = 0.015)	35.0 ± 3.3 Diff: −2.0 (*P* = 0.018)
	RVD(mm)	32.6 ± 3.0 Diff: −1.8 (*P* = 0.022)	32.5 ± 3.1 Diff: −1.7 (*P* = 0.025)
	EF(%)	54.5 ± 8.9 Diff: 1.2 (*P* = 0.35)	54.3 ± 9.1 Diff: 1.1 (*P* = 0.38)
	Categorical Variables	Positive Rate or OR (*P*-value)	Positive Rate or OR (*P*-value)
	AF(Yes?)	49.5%; OR = 1.32 (*P* = 0.12)	49.0%; OR = 1.30 (*P* = 0.13)
	PASP (Grade)	11.0%; OR = 0.85 (*P* = 0.40)	10.8%; OR = 0.83 (*P* = 0.42)
96 Months	Continuous Variables		
	MVPG (mmHg)	16.8 ± 5.8 Diff: −1.1 (*P* = 0.15)	16.7 ± 5.9 Diff: −1.0 (*P* = 0.18)
	MV(m/s)	2.19 ± 0.48 Diff: −0.08 (*P* = 0.18)	2.18 ± 0.47 Diff: −0.07 (*P* = 0.21)
	PHT (ms)	110 ± 32 Diff: −7.5 (*P* = 0.09)	109 ± 31 Diff: −7.2 (*P* = 0.11)
	TR area (cm^2^)	4.89 ± 2.15 Diff: −2.32 (*P* < 0.001)	4.85 ± 2.11 Diff: −2.28 (*P* < 0.001)
	RAD(mm)	36.7 ± 3.8 Diff: −2.4 (*P* = 0.008)	36.6 ± 3.7 Diff: −2.3 (*P* = 0.009)
	RVD(mm)	33.1 ± 3.5 Diff: −2.1 (*P* = 0.012)	33.0 ± 3.6 Diff: −2.0 (*P* = 0.015)
	EF(%)	53.5 ± 9.5 Diff: 0.8 (*P* = 0.52)	53.4 ± 9.6 Diff: 0.7 (*P* = 0.55)
	Categorical Variables		
	AF(Yes?)	53.0%; OR = 1.45 (*P* = 0.06)	52.5%; OR = 1.43 (*P* = 0.07)
	PASP (Grade)	16.3%; OR = 0.91 (*P* = 0.65)	16.0%; OR = 0.89 (*P* = 0.67)

Diff: Between-group difference (Group-A vs. Group-B).

OR, Odds Ratio for the treatment group.

Consistency Assessment: Based on the identical direction of effect, comparable magnitude of estimates (all differences <5%), and consistent statistical significance (or non-significance) between the two methods.

### Mid- to long-term tricuspid regurgitation outcomes

A total of 41 matched pairs completed long-term echocardiographic follow-up. There were no significant group differences in residual or recurrent tricuspid regurgitation at 6 and 24 months (all *P* > 0.05). However, the autologous pericardial soft ring group demonstrated significantly lower recurrence rates of moderate-or-greater tricuspid regurgitation at 48 and 96 months compared with the prosthetic ring group (all *P* < 0.05) (Table 3).

During the entire follow-up period, no patient in either group underwent surgical reintervention for recurrent tricuspid regurgitation.

### Right heart remodelling

Compared with the prosthetic ring group, the autologous pericardial soft ring group demonstrated significantly smaller right atrial diameter at 48 months (*P* < 0.05). No significant differences were observed between the two groups in right ventricular diameter at 96 months (*P* > 0.05) (Table 6).

**Table 6:** Comparison of postoperative follow-up examination indicators.

The Indicators	Group A	Group B	*P*
MVPG, mmHg			
6 months(*n* = 41)	14.56 ± 4.25	13.24 ± 4.07	0.078
24 months (*n* = 41)	15.68 ± 0.70	14.84 ± 0.61	0.188
48 months (*n* = 34)	15.33 ± 0.77	15.32 ± 0.82	0.498
96 months (*n* = 20)	16.29 ± 1.05	16.11 ± 0.74	0.447
MV, m/s			
6 months (*n* = 41)	1.95 ± 0.28	1.93 ± 0.54	0.425
24 months (*n* = 41)	2.07 ± 0.12	2.09 ± 0.12	0.449
48 months (*n* = 34)	1.89 ± 0.05	2.09 ± 0.12	0.064
96 months (*n* = 20)	2.02 ± 0.11	2.24 ± 0.18	0.158
PHT, ms			
6 months (*n* = 41)	98.49 ± 29.83	96.68 ± 22.73	0.379
24 months (*n* = 41)	97.42 ± 3.98	103.73 ± 4.70	0.170
48 months (*n* = 34)	98.22 ± 2.58	106.64 ± 5.51	0.074
96 months (*n* = 20)	103.44 ± 3.73	174.64 ± 65.20	0.138
PASP ≥ moderate (n, %)			
6 months (*n* = 41)	4, 9.75%	4, 9.75%	0.132
24 months (*n* = 41)	2, 4.88%	5, 12.20%	0.063
48 months (*n* = 34)	9, 26.47%	10, 29.41%	0.206
96 months (*n* = 20)	6, 30.00%	6, 30.00%	0.422
Atrial Fibrillation/Flutter (*n*, %)			
6 months (*n* = 41)	23, 56.10%	25, 61.00%	0.607
24 months (*n* = 41)	14, 34.14%	25, 60.98%	0.019
48 months (*n* = 34)	13, 38.24%	21, 61.76%	0.220
96 months (*n* = 20)	9, 45.00%	13, 65.00%	0.125
TR (cm^2^)			
6 months (*n* = 41)	1.81 ± 3.03	1.16 ± 1.72	0.117
24 months (*n* = 41)	2.32 ± 0.49	2.91 ± 0.37	0.173
48 months (*n* = 34)	2.88 ± 0.29	4.16 ± 0.36	0.004
96 months (*n* = 20)	3.55 ± 0.43	5.12 ± 0.52	0.010
RAD (mm)			
6 months (*n* = 41)	32.22 ± 4.45	33.71 ± 4.91	0.077
24 months (*n* = 41)	34.21 ± 0.93	35.83 ± 0.59	0.075
48 months (*n* = 34)	34.10 ± 0.98	36.31 ± 0.68	0.031
96 months (*n* = 20)	35.92 ± 1.03	36.88 ± 0.78	0.227
RVD (mm)			
6 months (*n* = 41)	29.39 ± 3.23	31.00 ± 3.57	0.018
24 months (*n* = 41)	31.15 ± 0.59	32.99 ± 0.53	0.013
48 months (*n* = 34)	31.35 ± 0.67	33.73 ± 0.59	0.004
96 months (*n* = 20)	32.95 ± 0.79	33.69 ± 1.04	0.283

## Discussion

### Risk factors and pathophysiology of FTR

Pulmonary hypertension and atrial fibrillation (AF) are major determinants of functional tricuspid regurgitation (FTR) progression (12–17). Elevated pulmonary artery systolic pressure (PASP) increases RV afterload, resulting in right-sided chamber dilation, papillary muscle displacement, and leaflet malcoaptation (17). AF contributes to atrial dilation and fibrosis, leading to tricuspid annular enlargement and worsening FTR (14–16). Progressive FTR further depresses RV function and is associated with reduced long-term survival (17).

Rheumatic heart disease (RHD) remains a major cause of pulmonary hypertension and AF in developing regions (18, 19). Persistent pulmonary hypertension after mitral valve surgery occurs frequently in RHD patients and adversely affects outcomes (20, 21). Additionally, prosthesis–patient mismatch (PPM) after mitral valve replacement may impair hemodynamics and perpetuate pulmonary hypertension, predisposing to postoperative TR progression (22, 23).

Given that PASP, AF, and PPM have substantial impacts on valve function recovery after surgery, we applied propensity score matching (PSM) to minimize their confounding effects and ensure comparability between groups.

### Efficacy and durability of annuloplasty techniques

Rigid or semi-rigid prosthetic ring annuloplasty (A-TVP) is commonly preferred in the surgical treatment of FTR due to its lower recurrence rate compared with suture-based techniques (7, 24, 25). However, prosthetic rings may restrict physiologic annular motion and increase the risk of conduction disturbances or ring-related complications such as coronary artery injury or endocarditis (26, 27).

In this study, P-TVP showed comparable early outcomes but significantly reduced long-term recurrence of ≥moderate TR and promoted more favorable right atrial reverse remodeling. The observed long-term benefits of P-TVP over A-TVP may be attributable to its flexible biomechanical properties, which better preserve annular dynamics and valvular complex integrity (28–31). Unlike rigid or semi-rigid rings that statically fix the annulus, a soft biological ring permits adaptive changes in annular shape and size throughout the cardiac cycle and during ventricular remodeling, potentially sustaining more effective leaflet coaptation over time. This hypothesis is supported by earlier imaging studies demonstrating greater annular area reduction and preserved saddle-shaped motion with flexible vs. rigid annuloplasty (26, 32). Autologous tissue may also facilitate biological integration and adaptive annular remodeling (33). However, because advanced imaging modalities such as 3D echocardiography or cardiac magnetic resonance were not used to directly assess annular dynamics in this study, these mechanistic explanations remain speculative and warrant confirmation in future dedicated investigations.

Moreover, improved RV–annular interaction may facilitate more effective reverse remodeling following correction of left-sided lesions (32, 33).

Although additional mechanistic studies are required, the biological compatibility and adaptive compliance of autologous tissue could contribute to enhanced durability. Further long-term and multicenter follow-up is warranted to confirm our results beyond 10 years.

### Divergence in atrial fibrillation prevalence

An interesting finding was the significantly lower incidence of AF in the P-TVP group compared to the A-TVP group at 24-month follow-up (Table 4). Although the exact mechanism cannot be elucidated from our data, we speculate that it may be related to differential effects on right atrial pathophysiology. The flexible pericardial ring may allow more physiological atrial contraction and reduce chronic tension on the right atrial wall and interatrial septum, thereby creating a less arrhythmogenic substrate. In contrast, a rigid prosthetic ring could impose a fixed geometry that might compromise optimal atrial mechanics and promote fibrosis. Additionally, this difference could be influenced by patient compliance with postoperative AF medication, which was not systematically recorded in our retrospective data. This observation warrants further investigation, as a reduction in AF burden may represent an additional clinical benefit of biological annuloplasty.

### Clinical implications

This propensity-matched retrospective study provides preliminary evidence that autologous pericardial soft-ring annuloplasty may be associated with better long-term tricuspid regurgitation control and right atrial reverse remodeling compared with prosthetic ring annuloplasty in patients with rheumatic heart disease. However, given the modest sample size, incomplete long-term follow-up, and single-center design, these findings should be regarded as hypothesis-generating rather than conclusive. The proposed mechanistic advantages—including preserved annular dynamics and biological integration—remain speculative and require direct validation using advanced imaging techniques.

Future prospective, multicenter studies with standardized echocardiographic protocols, comprehensive right heart function assessment, and rigorous follow-up retention strategies are essential to determine whether this technique confers durable clinical benefit over existing approaches.

### Limitations

This study has several limitations. First, as a retrospective analysis, unmeasured confounding factors may still exist despite the use of propensity score matching to reduce baseline selection bias, and the matching process inevitably resulted in the exclusion of eligible cases, which may limit generalizability. Second, although multiple imputations were utilized to address missing follow-up data, the method relies on the assumption that data were missing completely at random and may not fully reflect real-world conditions. Third, this was a single-center study with a relatively limited postoperative follow-up cohort and a progressive decline in sample size over longer follow-up intervals. Fourth, echocardiographic assessment primarily relied on two-dimensional parameters, without advanced right-heart functional evaluation such as 3D imaging or cardiac magnetic resonance. Fifth, while follow-up data included parameters related to mitral stenosis (e.g., MVPG, MV, PHT) and a preliminary discussion of prosthesis-patient mismatch (PPM) was provided, detailed characterization of aortic valve status and mitral regurgitation was lacking. This limitation may affect the interpretation of our findings and should be acknowledged. Future prospective studies are warranted to comprehensively evaluate these concomitant valvular conditions. Sixth, the study spanned a 8-year period, during which surgical experience and team proficiency with the P-TVP technique inevitably evolved. Although the core surgical team remained largely stable and the operative technique was standardized early in the series, a potential learning curve effect cannot be entirely excluded and may have influenced early outcomes. However, the consistent long-term benefits observed across the study period argue against a major confounding effect of experience alone.

Future prospective, multicenter studies with standardized imaging and larger long-term cohorts are warranted to validate the durability and clinical applicability of autologous pericardial soft ring annuloplasty.

#### Attrition in long-term follow-up

**Figure 2:**
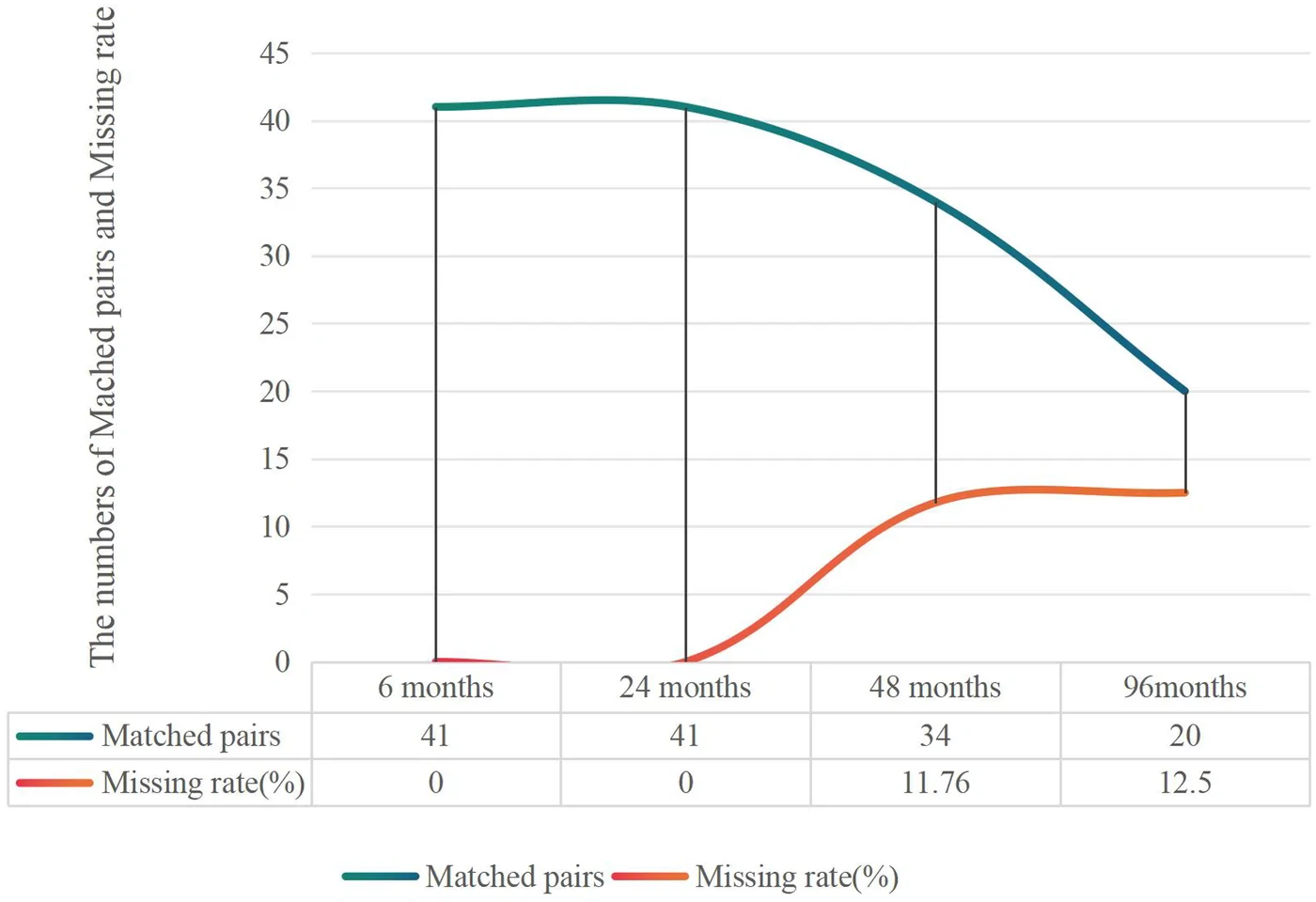
Changes in matched cohort size and missing data over time.

A notable limitation of this study is the progressive decline in the number of patients with complete echocardiographic data over time. As shown in Figure 2, the number of matched cases decreased from 41 pairs at 48 months to 34 pairs, with a missing data proportion of 11.76%. By 96 months, the matched cases further declined to 20 pairs, corresponding to a missing data proportion of 12.50%., primarily due to loss to follow-up resulting from relocation, changes in contact information, and non-response—a common issue in long-term surgical research. The reduced sample size diminishes statistical power for detecting differences at later time points and increases the risk of type II error. The potential impact of attrition on the stability of long-term effect estimates and the generalizability of the findings should be acknowledged. Future prospective studies should implement dedicated follow-up protocols to minimize participant loss.

## Conclusions

In this retrospective study, autologous pericardial soft ring annuloplasty demonstrated comparable early outcomes but superior long-term durability compared with prosthetic ring repair, particularly in reducing recurrent functional tricuspid regurgitation and promoting favorable right-sided cardiac remodeling. These findings suggest that P-TVP, with its physiological annular flexibility and biological integration, may preserve right ventricular systolic-diastolic function and restore tricuspid valvular complex integrity. The unique histological features of the autologous pericardium, combined with continuous suture fixation, may synergistically maintain the three-dimensional geometry of the tricuspid annulus and enhance biomechanical support, thereby contributing to reduced late recurrence.

Future research should incorporate multimodal imaging (e.g., three-dimensional echocardiography and cardiac magnetic resonance) and quantitative assessments of RV function (e.g., TAPSE, FAC, RV strain) to further clarify the mechanisms of reverse remodeling. Prospective multicenter studies with larger samples and longer follow-up durations are warranted to validate the long-term clinical applicability of this technique.

## Data Availability

The original contributions presented in the study are included in the article/Supplementary Material, further inquiries can be directed to the corresponding author.
