# Correction: Autologous pericardial soft-ring annuloplasty may offer improved long-term control of rheumatic functional tricuspid regurgitation: a propensity-matched cohort study

**DOI:** 10.3389/fcvm.2026.1954100

**Published:** 2026-08-25

**Authors:** Sicong Li, Wei Jiang, Yuyao Qin, Kequan Wei, Hui Chen, Songtao Liu, Xiaomao Long

**Affiliations:** Department of Cardiovascular Surgery, People's Hospital of Guangxi Zhuang Autonomous Region, Nanning, Guangxi Zhuang Autonomous Region, China

**Keywords:** functional tricuspid regurgitation, pericardial annuloplasty, propensity score matching, prosthetic ring, rheumatic heart disease

The affiliation for all authors was erroneously published as:

Department of Cardiovascular Surgery, People's Hospital of Guangxi Zhuang Autonomous Region, Guangxi Medical University, Nanning, Guangxi Zhuang Autonomous Region, China

The correct affiliation is:

Department of Cardiovascular Surgery, People's Hospital of Guangxi Zhuang Autonomous Region, Nanning, Guangxi Zhuang Autonomous Region, China

The original version of this article has been updated.

